# The characteristics of bacterial nanocellulose gel releasing silk sericin for facial treatment

**DOI:** 10.1186/s12896-014-0104-x

**Published:** 2014-12-09

**Authors:** Pornanong Aramwit, Nipaporn Bang

**Affiliations:** Bioactive Resources for Innovative Clinical Applications Research Unit and Department of Pharmacy Practice, Faculty of Pharmaceutical Sciences, Chulalongkorn University, PhayaThai Road, Phatumwan, Bangkok 10330 Thailand

**Keywords:** Bacterial nanocellulose, *Gluconacetobacter xylinus*, Coconut water, Silk sericin, Facial mask

## Abstract

**Background:**

Recently, naturally derived facial masks with beneficial biological properties have received increasing interest. In this study, silk sericin-releasing bacterial nanocellulose gel was developed to be applied as a bioactive mask for facial treatment.

**Results:**

The silk sericin-releasing bacterial nanocellulose gel produced at a pH of 4.5 had an ultrafine and extremely pure fiber network structure. The mechanical properties and moisture absorption ability of the gel were improved, compared to those of the commercially available paper mask. Silk sericin could be control-released from the gel. A peel test with porcine skin showed that the gel was less adhesive than the commercially available paper mask, which would be removed from the face more easily without pain. The *in vitro* cytotoxicity test showed that the gel was not toxic to L929 mouse fibroblast and HaCaT human keratinocyte cells. Furthermore, when implanted subcutaneously and evaluated according to ISO10993-6 standard, the gel was not irritant to tissue.

**Conclusion:**

The silk sericin-releasing bacterial nanocellulose gel had appropriate physical and biological properties and safety for the facial treatment application.

## Background

Nowadays, various kinds of facial masks are applied for different purposes: for example, revitalizing, healing, and refreshing, and may yield other temporary or long-term benefits to facial skin. Recently, searching for a new naturally derived facial mask with beneficial biological properties has become highly competitive. Cellulose is an organic polysaccharide consisting of a linear chain of several hundred to over ten thousand β (1 → 4) linked D-glucose units [1]. Cellulose is frequently derived from plant sources; however, it can also be synthesized by a variety of microorganisms such as bacteria, algae, and fungi [2]. Biosynthetic bacterial cellulose shows appealing characteristics of high purity and unique physicochemical properties such as high tensile strength, crystallinity, and water absorption capacity [3]. *Gluconacetobacter xylinus* (formerly known as *Acetobacter xylinum*) is a species of gram-negative bacteria that can produce high amounts of cellulose. Glucose was used as the common substrate for the bacterial nanocellulose produced by *G. xylinus* [4]. Therefore, searching for cost-effective sources of glucose that can form bacterial nanocellulose with maintained high purity and quality is important.

Coconut is a fruit grown in tropic and subtropic areas that is known for its great versatility, as seen in the many domestic, commercial, and industrial uses of its different parts. Coconut contains a large quantity of coconut water, which is mostly discarded as waste from various agro industries. Coconut water is rich in sucrose (sugar) and nitrogen-containing compounds. Therefore, it could be a cheap substrate for the production of bacterial nanocellulose [5].

In this study, the bacterial nanocellulose was produced at the surface of coconut water and other suitable media by a gram-negative rod-shaped bacterium, *G. xylinus.* The fermentation was carried out in static cultures at 30°C in acidic pH varying from 3 to 5. We investigated the effect of the processing pH on the formation of bacterial nanocellulose gel. As the aim was to be applied as a bioactive facial mask, our bacterial nanocellulose gel was adsorbed with silk sericin. Silk sericin is a biocompatible protein derived from the silkworm cocoon. The biological roles of silk sericin such as an antioxidant, bioadhesive, and bioactive agent, as well as a promising implant for tissue-supporting prosthetics for the human body, have been reported [6,7]. Silk sericin could also activate collagen production and significantly increase epithelialization in wounds [8-10]. Herein, the physical properties of the silk sericin-releasing bacterial nanocellulose gel produced at optimal pH were characterized. A test of the release of silk sericin from the gel was carried out. In addition, *in vitro* cytotoxicity and *in vivo* safety tests according to ISO 10993–6 standards were performed, in comparison to the commercially available paper mask (Facial Paper Mask, Hefei Wenqi Industrial and Trading, Co., Ltd., Anhui, Republic of China). It is hypothesized that silk sericin-releasing bacterial nanocellulose gel would be a good candidate as a bioactive facial mask for facial treatment. Silk sericin released from bacterial nanocellulose gel may help to revitalize, heal, or refresh the skin.

## Results and discussion

### Physical appearance and structure of the bacterial nanocellulose gel

**Figure 1:**
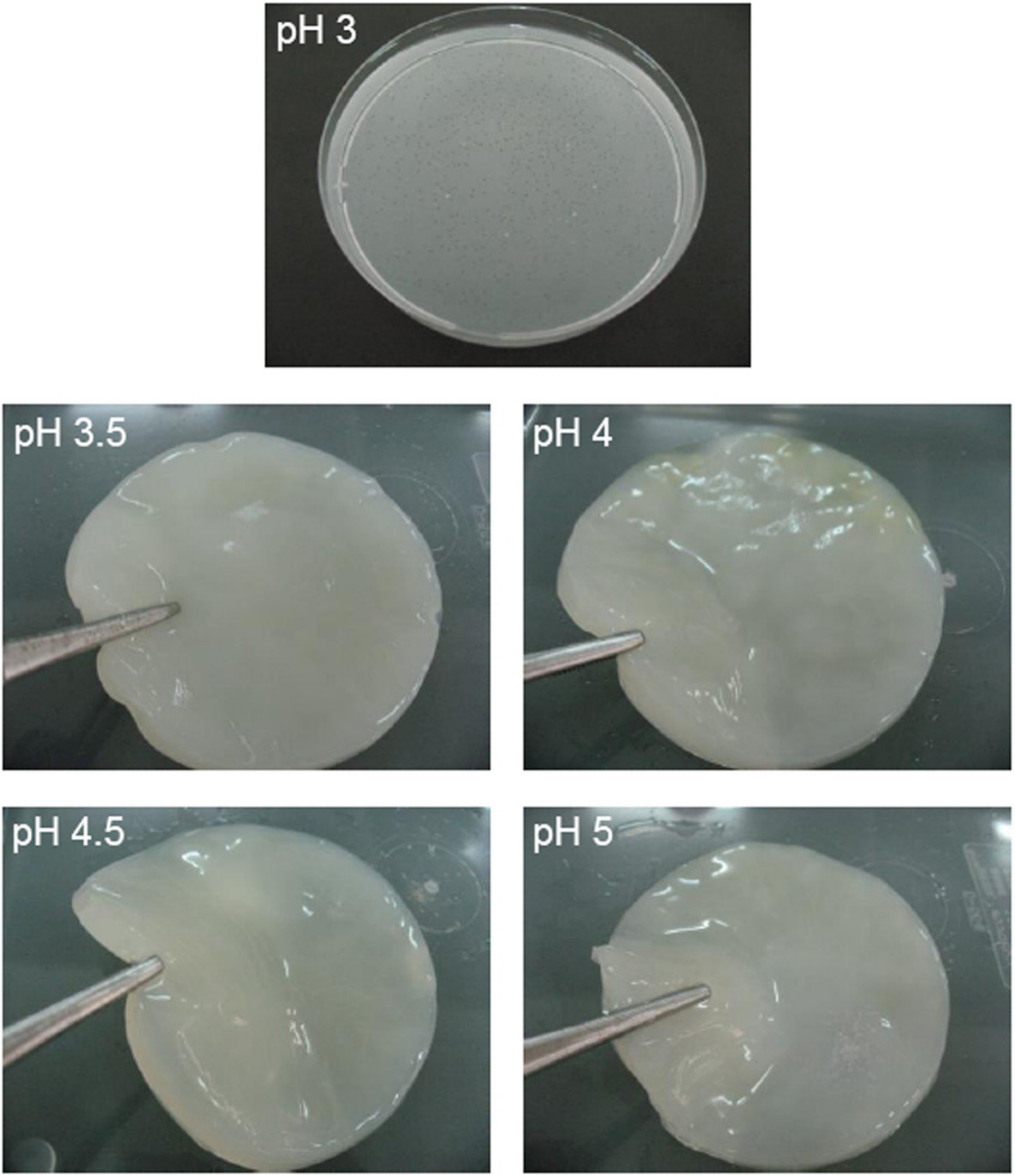
**Physical appearance of the bacterial nanocellulose gels prepared at different pH (3, 3.5, 4, 4.5, and 5).**

**Figure 2:**
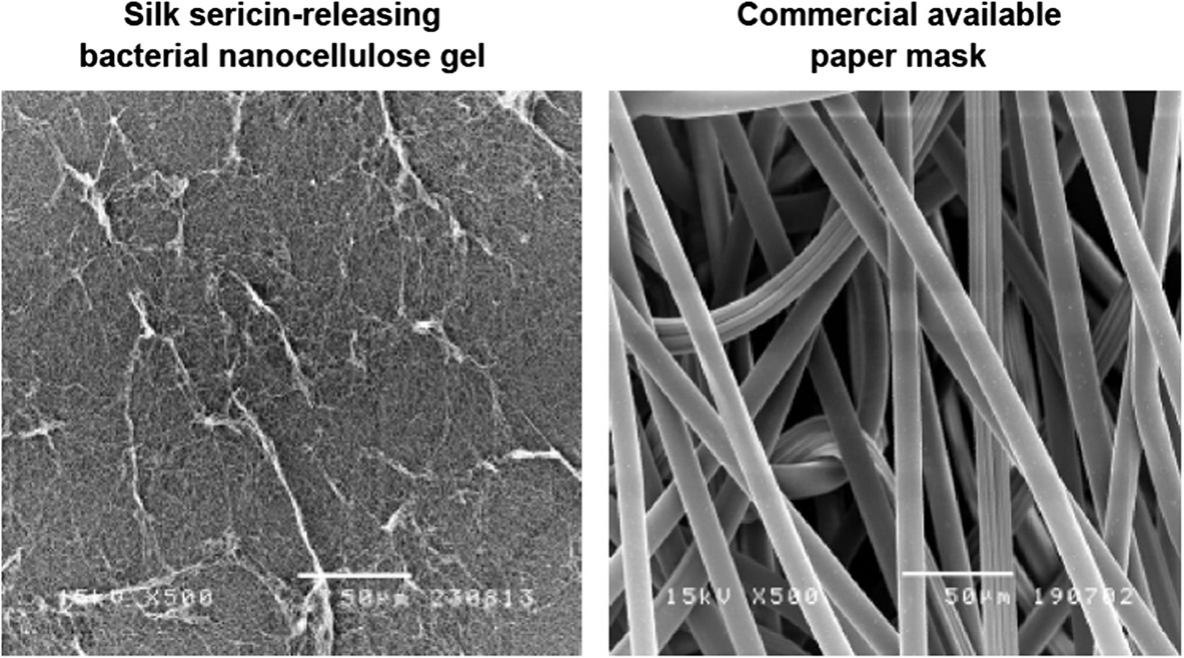
**SEM micrographs of the silk sericin-releasing bacterial nanocellulose gel and the commercial available paper mask (scale bar = 50 μm).**

We first studied the influence of pH condition on the formation of bacterial nanocellulose gel because it was supposed that pH may affect the rate of bacterial growth, which was important for the formation of bacterial nanocellulose gel. The bacterial nanocellulose gels prepared from various pH had different physical appearances, as shown in Figure 1. The gels could be set up at a pH of 3.5 to 5 but did not form at a pH of 3. Table 1 summarizes the physical appearance and thickness of the bacterial nanocellulose gels prepared at different pH. At a pH of 3.5 and 4, hard and stable gels with a smooth surface were produced. The gel produced at a pH of 4.5 was also stable with a smooth surface but softer. In contrast, the production at a pH of 5 resulted in a very soft gel. The thickness of bacterial nanocellulose gels seemed to decrease with the increasing pH. The processing pH at 3.5 produced a 0.7 cm gel while the pH at 5 produced a 0.3 cm gel. A pH of 4.5 would be the optimal condition for bacterial growth, as previously reported by Embuscado *et al*. [3]. The 0.6 cm soft and stable gel with a smooth surface produced at a pH of 4.5 was selected for further investigation in this study. To improve the biological properties, the bacterial nanocellulose gel prepared at a pH of 4.5 was absorbed with silk sericin solution to obtain the silk sericin-releasing bacterial nanocellulose gel. It is well known that silk sericin has many biological roles such as an antioxidant, bioadhesive, and bioactive activities. It could also activate collagen production and epithelialization, which would be beneficial for facial treatment application [6-10]. The structure of silk sericin-releasing bacterial nanocellulose gel was observed in comparison to the commercially available paper mask, as displayed in Figure 2. We found that the silk sericin-releasing bacterial nanocellulose gel showed a highly fibrous structure with very fine fibers, which corresponded to the report of Vandamme *et al*. [11] that the bacterial cellulose usually has an ultrafine and extremely pure fiber network structure. On the other hand, the cotton fibers of the commercially available paper mask were much larger (~10 μm in diameter).

**Table 1:** **Physical appearance and thickness of the bacterial nanocellulose gels prepared at different pH**

**Processing pH**	**Physical appearance of the nanocellulose gel**	**Thickness (cm)**
3	Not formed	-
3.5	Hard and stable gel with smooth surface	0.7
4	Hard and stable gel with smooth surface	0.5
4.5	Soft and stable gel with smooth surface	0.6
5	Very soft gel with smooth surface	0.3

### Mechanical properties of the silk sericin-releasing bacterial nanocellulose gel

To be applied as a facial mask, the silk sericin-releasing bacterial nanocellulose gel should have appropriate mechanical properties. For example, it should be flexible, stretchable, and elongatable in order to adhere completely to the whole of the facial skin. The tensile modulus and percentage of elongation of the dried and wet silk sericin-releasing bacterial nanocellulose gel, compared with those of the commercially available paper mask, are demonstrated in Table 2. Both in the dry and wet conditions, the tensile modulus of the silk sericin-releasing bacterial nanocellulose gel were significantly higher than that of the commercially available paper mask. The percentage of elongation of both samples in the dry state was the same. However, in the wet state, the silk sericin-releasing bacterial nanocellulose gel had a significantly higher elongation percentage than the commercially available paper mask. This could be explained by the fact that our gel is a bacterial nanocellulose, which has more crystalline structure and forms characteristic ribbon-like microfibrils, compared to the plant cellulose of a paper mask [12]. In addition, the silk sericin absorbed on the fibers of our bacterial nanocellulose gel would improve the mechanical properties. Improvement of the mechanical properties by surface medication has been reported elsewhere [13,14].

**Table 2:** **Mechanical properties of the silk sericin-releasing bacterial nanocellulose gel and the commercial available paper mask at dry and wet conditions**

	**Tensile modulus (N/mm** ^**2**^ **)**		**Elongation (%)**	
	**Dry**	**Wet**	**Dry**	**Wet**
Silk sericin-releasing bacterial nanocellulose gel	30.84 ± 6.99*	11.23 ± 1.26*	32.89 ± 11.89	67.03 ± 5.72*
Commercial available paper mask	0.86 ± 0.22	0.87 ± 0.05	21.88 ± 8.09	26.9 ± 4.38

*, *p* < 0.05 significant against the value of commercial available paper mask at corresponding condition.

### Moisture absorption capability of the silk sericin-releasing bacterial nanocellulose gel

**Figure 3:**
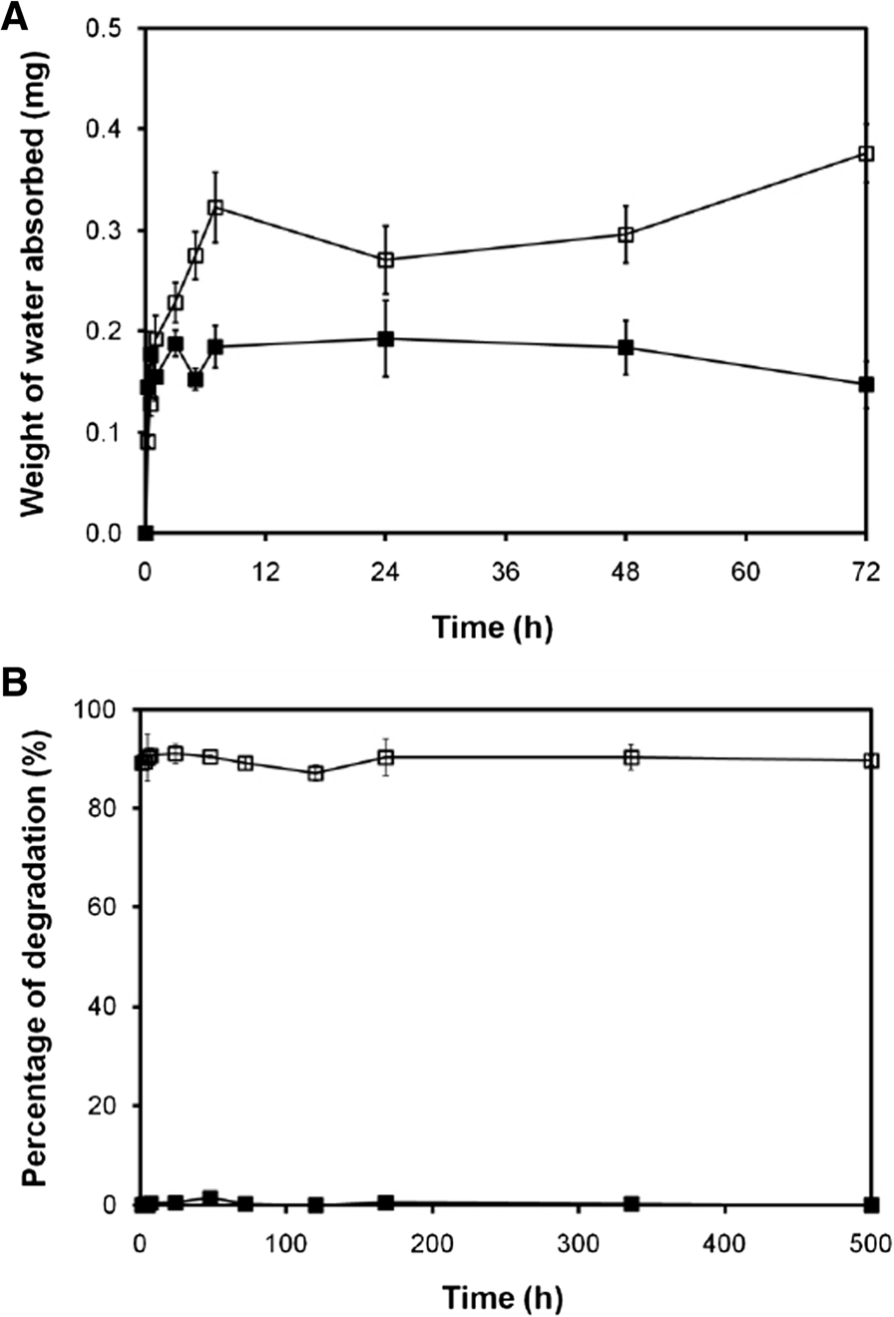
**Moisture absorption and enzymatic biodegradation properties of biocelllose gel and paper mask. (A)** Weight of water absorbed on the samples after placing in humidified desiccators with a controlled relative humidity at 81.47 ± 1.48%, 25°C for different time periods. **(B)** Percentage of degradation of the samples after incubated in 1.6 μg/ml lysozyme solution (pH 7.4) at 37°C for different time periods. (**□**) silk sericin-releasing bacterial nanocellulose gel and (■) the commercial available paper mask.

The facial mask should have good water absorption ability in order to maintain the moisture within its structure during the treatment. Figure 3A shows the moisture absorption capability of the silk sericin-releasing bacterial nanocellulose gel, compared to that of the commercially available paper mask. Initially, the amount of water absorbed on the silk sericin-releasing bacterial nanocellulose gel was increased over time and became stable thereafter. The silk sericin-releasing bacterial nanocellulose gel showed higher moisture absorption capability than the commercially available paper mask during the incubation period. This was due to the high hydrophilicity of both bacterial nanocellulose and silk sericin [15,16].

### Enzymatic biodegradation rate of the silk sericin-releasing bacterial nanocellulose gel

The biodegradability of a facial mask indicates that it is bioenvironmentally friendly. The percentage of degradation of the silk sericin-releasing bacterial nanocellulose gel, compared to that of the commercially available paper mask, is illustrated in Figure 3B. A total of 90% of the silk sericin-releasing bacterial nanocellulose gel was degraded during the incubation period. On the other hand, the commercially available paper mask was not degraded in the same condition. This would be explained by the fact that our bacterial nanocellulose gel was made of coconut water, bacteria, and silk sericin, which are all quickly biodegradable. In contrast, a commercially available paper mask is mainly cotton made of plant cellulose, waxes, and some fatty substances, which would be degraded more slowly. In addition, the plant bacterial nanocellulose of a paper mask has a mesh-like bulk work structure and tough property while our bacterial nanocellulose is more chemically pure and has a higher water-holding capacity and hydrophilicity. Thus, the bacterial nanocellulose would degrade faster than the plant bacterial nanocellulose [15].

### The *in vitro* release profile of silk sericin

**Figure 4:**
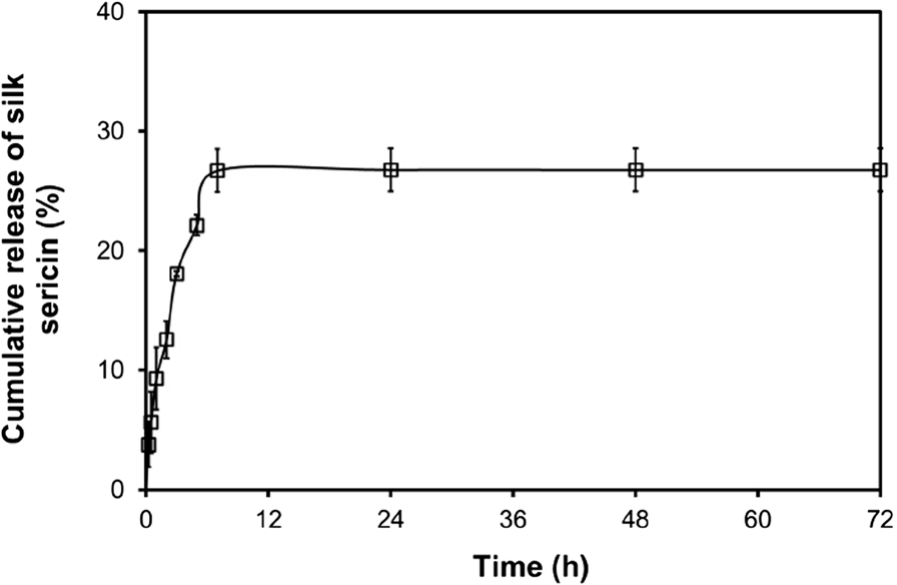
**Percentage of cumulative release of silk sericin from the silk sericin-releasing bacterial nanocellulose gel after incubated in PBS solution (pH 7.4) at 37°C for different time periods.**

We have supposed here that the release of silk sericin from the bacterial nanocellulose gel would support the facial treatment in terms of the activation of collagen production and epithelialization of the skin [8,9]. Figure 4 demonstrates the release profile of silk sericin from the silk sericin-releasing bacterial nanocellulose gel. Initially (0–7 h), silk sericin was burst released from the bacterial nanocellulose gel. Later on, the release of silk sericin from the bacterial nanocellulose gel reached the plateau at 27%. The release of silk sericin was possibly governed by two mechanisms: diffusion and material degradation [17,18]. The initial burst was the diffusional release of silk sericin absorbed on the fiber’s surface. Thereafter, the silk sericin interacted with the bacterial nanocellulose fibers that were released during the degradation of the fibers. We supposed that the controlled release behavior of silk sericin from the bacterial nanocellulose gel may be beneficial for facial treatment.

### Adhesive property of the silk sericin-releasing bacterial nanocellulose gel

**Figure 5:**
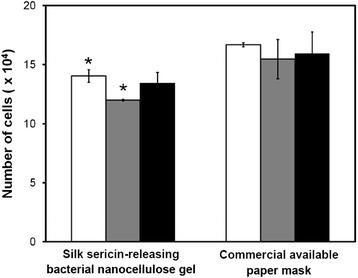
**Number of cells on the silk sericin-releasing bacterial nanocellulose gel and the commercial available paper mask after attached to full-thickness wound of porcine skin for 4 (□), 24 (gray square symbol), and 48 h (■).**

A good facial mask should be less adhesive in order to be removed from face easily without pain. The adhesive property of the silk sericin-releasing bacterial nanocellulose gel and the commercially available paper mask was evaluated from a number of cells on the samples after being attached to a wound on porcine skin, namely a peel assay. After being attached to the wound on porcine skin for 4 and 24 h, the number of cells found on the silk sericin-releasing bacterial nanocellulose gel was significantly lower than that found on the commercially available paper mask (Figure 5). Therefore, the silk sericin-releasing bacterial nanocellulose gel was less adhesive than the commercially available paper mask, which may be removed more easily and with reduced pain.

### Non-cytotoxicity of the silk sericin-releasing bacterial nanocellulose gel

**Figure 6:**
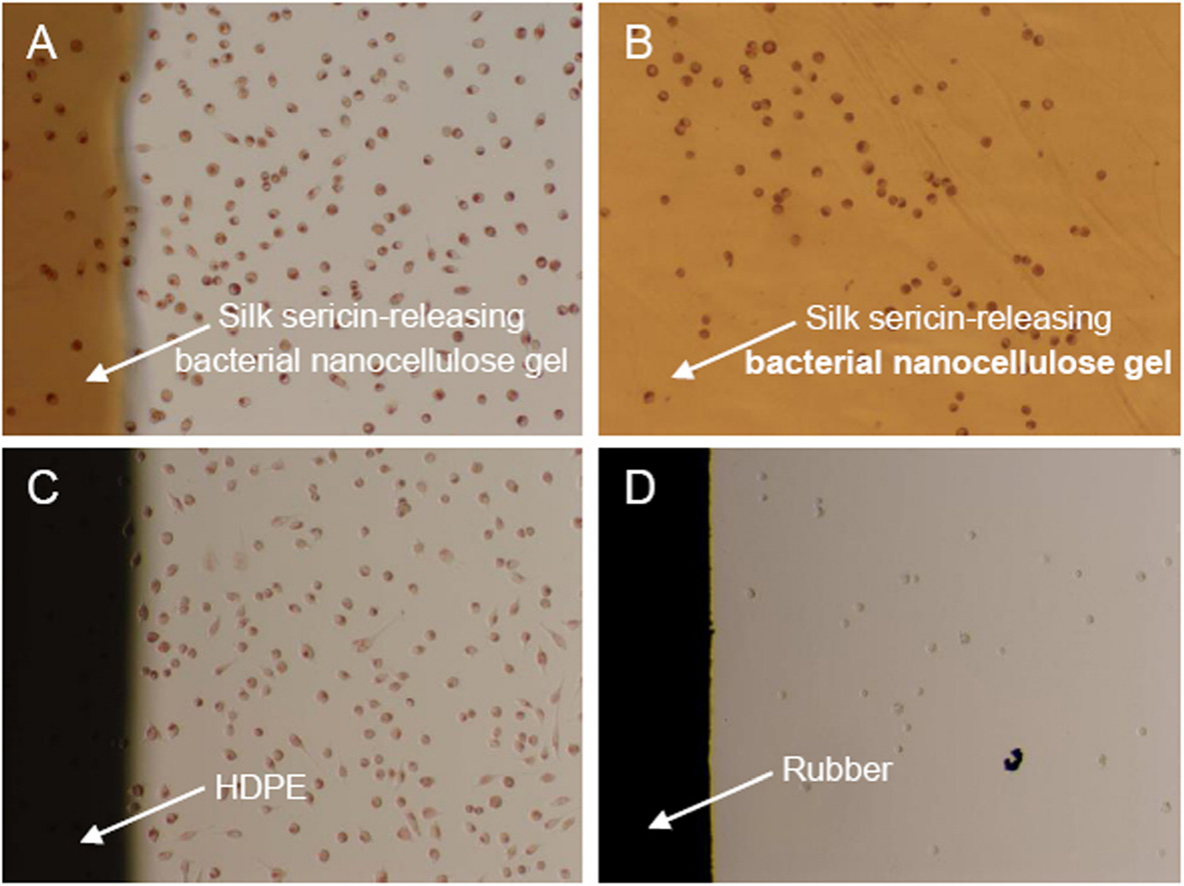
**Morphology of L929 mouse fibroblast cells after cultured on materials for 48 h. (A,B)** silk sericin-releasing bacterial nanocellulose gel, **(C)** high-density polyethylene (HDPE, negative control), and **(D)** rubber (positive control).

**Figure 7:**
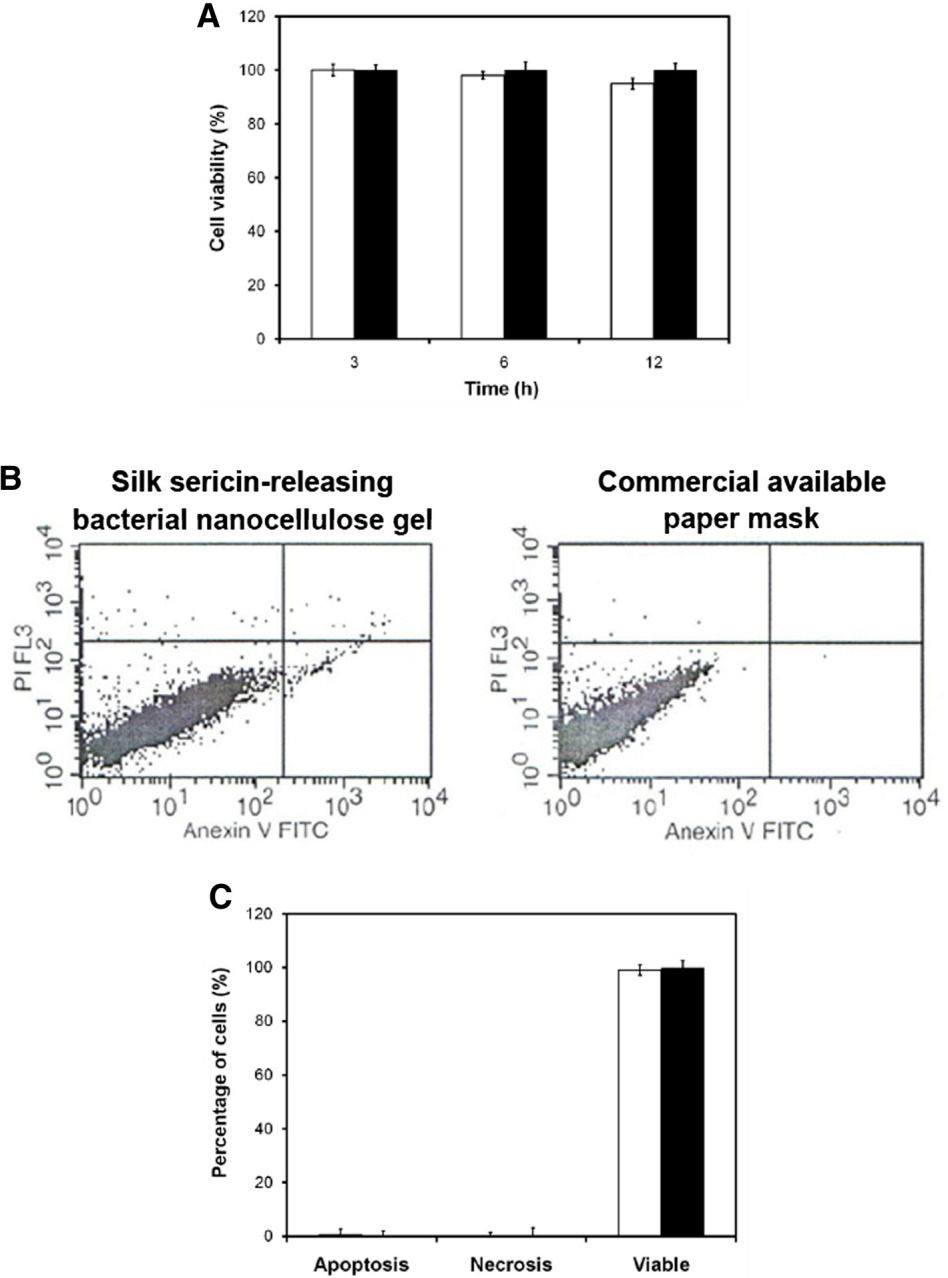
**Non-cytotoxicity of silk sericin-releasing bacterial nanocellulose gel and paper mask. (A)** Percentage of viability of HaCat cells cultured in the presence of samples for 3, 6, and 12 h. **(B)** Apoptotic profile and **(C)** Viability percentage of HaCat cells cultured in the presence of samples for 3 h, analyzed by Annexin V-FITC/PI double staining assay. (□) silk sericin-releasing bacterial nanocellulose gel (■) and the commercial available paper mask.

The silk sericin-releasing bacterial nanocellulose gel must not be cytotoxic when applied as a facial mask in direct contact with facial skin. After culture by a direct contact with the sample, the viable L929 cells were observed on the silk sericin-releasing bacterial nanocellulose gel, similar to those cultured on the HDPE as a negative control (Figures 6A–C). In contrast, the cells were dead when cultured on rubber as a positive control (Figure 6D). Furthermore, HaCat cells cultured in the presence of silk sericin-releasing bacterial nanocellulose gel for 3–12 h showed as high viability (~100%) as those cultured in the presence of a commercially available paper mask (Figure 7A). The apoptotic profile of HaCat cells cultured in the presence of the sample, as analyzed by Annexin V-FITC/PI double staining assay, showed that cells were both Annexin V-FITC- and PI-negative (Figure 7B). This indicated that most of the cells (~100%) were viable, but not in the apoptotic or necrotic state (Figure 7C). The results confirmed that our silk sericin-releasing bacterial nanocellulose gel was non-cytotoxic.

### *In vivo* safety of the silk sericin-releasing bacterial nanocellulose gel

**Figure 8:**
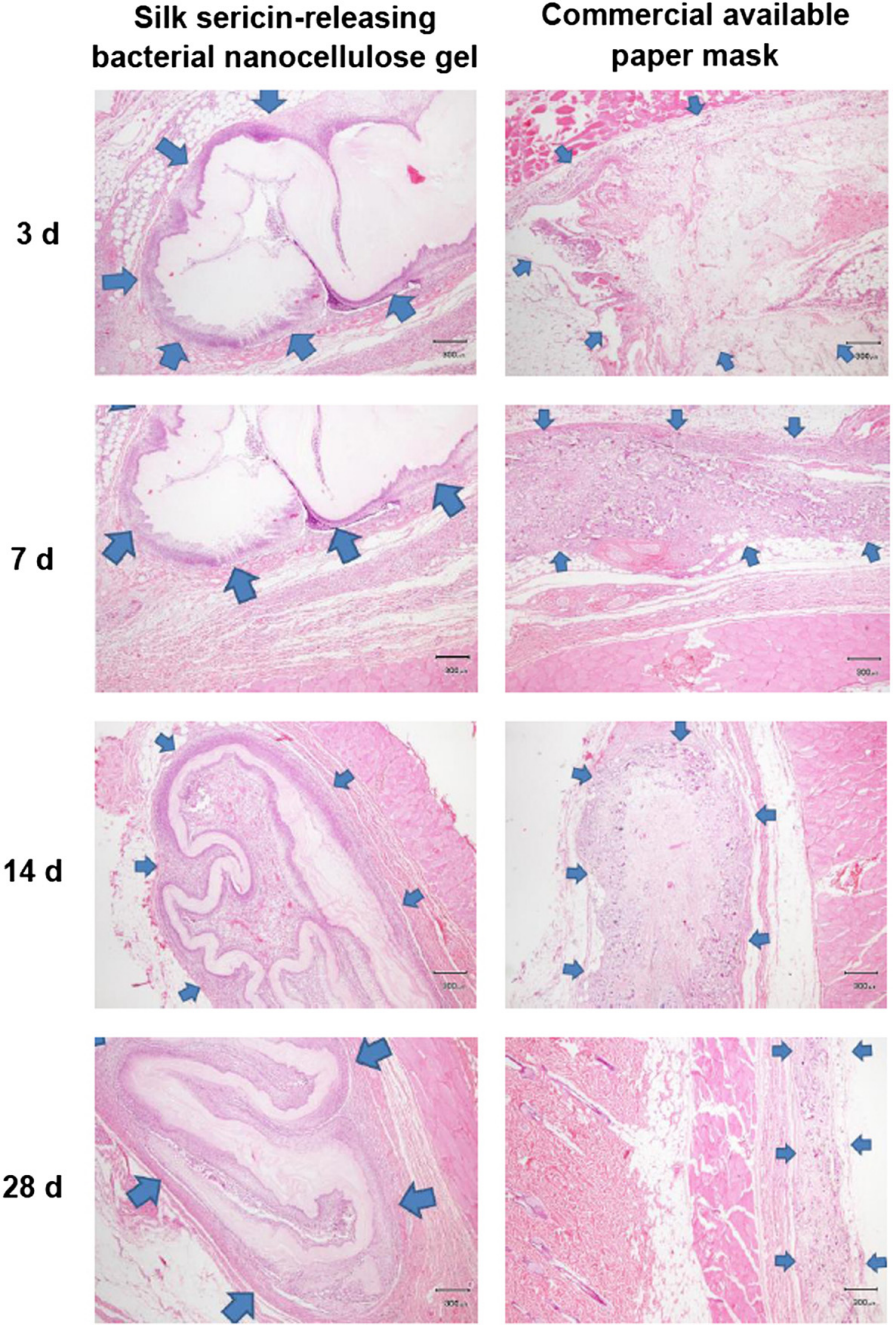
**Microscopic images of H&E-stained sections of silk sericin-releasing bacterial nanocellulose gel and the commercial available paper mask after subcutaneous implantation for 3, 7, 14, and 28 days.** Scale bar = 300 μm, arrow: interface between sample implanted and surrounding tissue.

**Figure 9:**
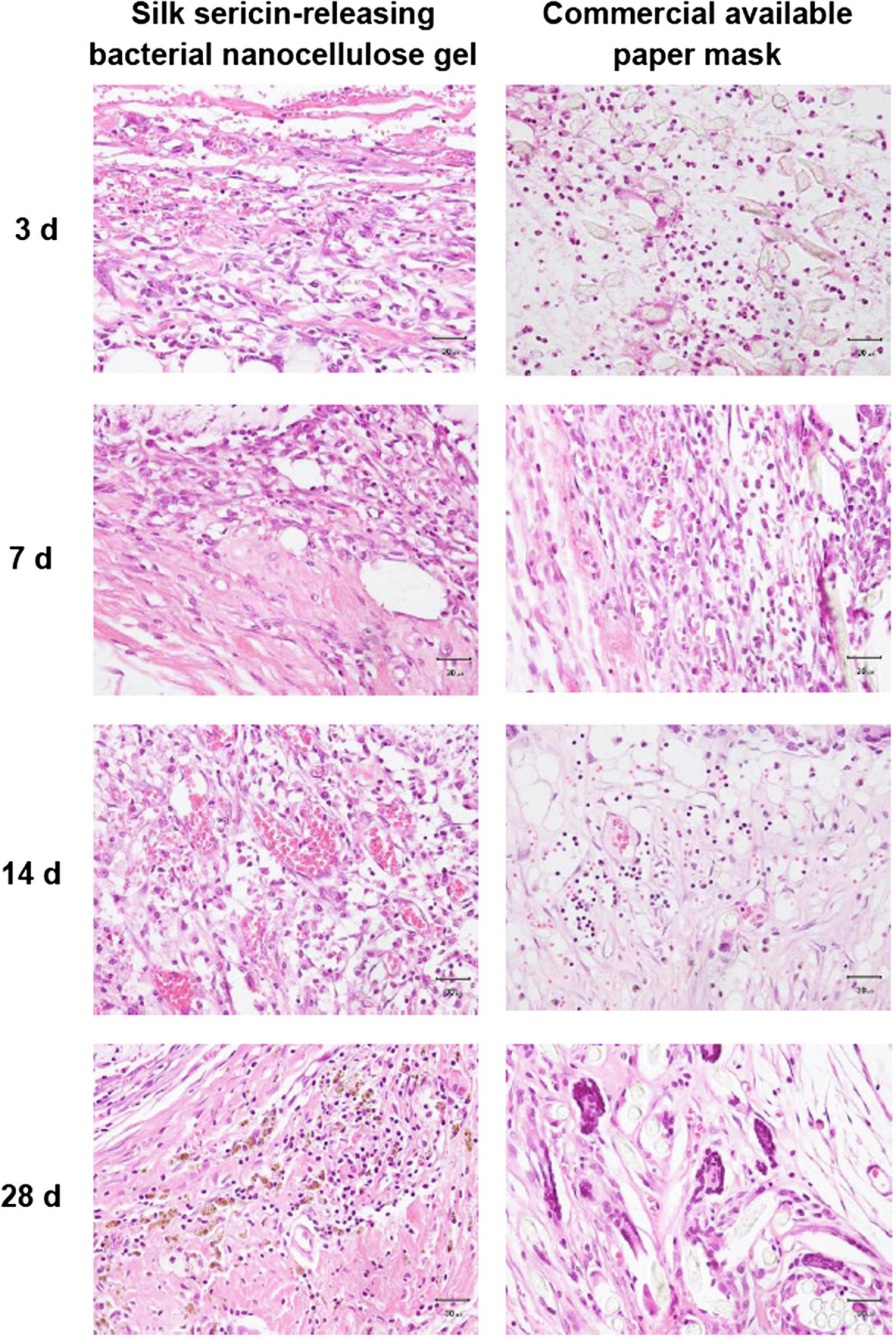
**Microscopic images of H&E-stained sections indicating inflammatory cells in silk sericin-releasing bacterial nanocellulose gel and the commercial available paper mask after subcutaneous implantation for 3, 7, 14, and 28 days.**

To prove its safety for cosmetic application, the silk sericin-releasing bacterial nanocellulose gel was subcutaneously implanted into rats and the tissue responses were evaluated according to ISO10993-6 standard (Biological evaluation of medical devices – Part 6: Tests for local effects after implantation) in comparison to the commercially available paper mask. The rats that received the implantation of both samples were healthy throughout the implantation period. No signs of inflammation (i.e. redness, swelling, pain, and heat) were observed in any of the rats. Figure 8 shows images of the H&E-stained sections of silk sericin-releasing bacterial nanocellulose gel and the commercially available paper mask implanted. The arrows indicate the interface between the implanted sample and surrounding tissue. No excessive inflammatory reaction was detected around the implantation sites. The infiltration of inflammatory cells into the implanted samples is shown in Figure 9. The number of inflammatory cells that infiltrated into the silk sericin-releasing bacterial nanocellulose gel was comparable to that of the commercially available paper mask during the implantation period. The intensity of inflammatory cells, necrosis, fibrosis, neovascularization, and fatty infiltrate was graded as presented in Table 3. It was observed that polymorphonuclear cells were packed and infiltrated into silk sericin-releasing bacterial nanocellulose gel after 3 and 7 weeks of implantation. However, its intensity was reduced thereafter. Lymphocytes, macrophages, and neovascularization were also found in both samples during the implantation period. The implanted commercially available paper mask showed more intensity of fatty infiltrate and giant cells than the implanted silk sericin-releasing bacterial nanocellulose gel. On the other hand, plasma cells and necrosis were not observed in either sample during the implantation period. To sum up, the implantation of silk sericin-releasing bacterial nanocellulose gel was evaluated as being nonirritant, relative to the commercially available paper mask. This confirmed that our silk sericin-releasing bacterial nanocellulose gel was safe for further investigation in the application of facial treatment.

**Table 3:** **Average intensity of inflammatory cells, necrosis, fibrosis, neovascularization, and fatty in silk sericin-releasing bacterial nanocellulose gel and the commercial available paper mask after subcutaneous implantation for 3, 7, 14, and 28 days**

	**3 days**		**7 days**		**14 days**		**28 days**	
	B*	C**	**B**	**C**	**B**	**C**	**B**	**C**
PMN	4 †	2.25	4	1.75	3.25	1.75	3.25	1
Lymphocytes	1	2	2	2	2.75	2	2.5	1.75
Plasma cells	0	0	0	0	0	0	0	0
Macrophages	3	2.5	3	3	3	3	3	3
Giant cells	0	0.5	0	2	0.75	3	1	3
Necrosis	0	0	0	0	0	0	0	0
Fibrosis	4	2.75	4	3.25	4	4	4	3.5
Neovascularization	3	2.5	3	1.25	2.75	1.75	2	3
Fatty infiltrate	0	3	0	1	0	0.75	0	0.5

*B: silk sericin-releasing bacterial nanocellulose gel.

**C: commercial available paper mask.

**†** Intensity 0–4: 0 = not observed, 1 = rare, 2 = minimal, 3 = heavily infiltrate, and 4 = packed infiltrate.

## Conclusion

Silk sericin-releasing bacterial nanocellulose gel has been developed in this study to be applied as a bioactive mask for facial treatment. We found that a pH of 4.5 was optimal for the production of the bacterial nanocellulose gel, resulting in a 0.6 cm soft and stable gel with a smooth surface. To improve the biological properties, the gel was absorbed with silk sericin. The silk sericin-releasing bacterial nanocellulose gel had an ultrafine and extremely pure fiber network structure. The mechanical properties and moisture absorption ability of the silk sericin-releasing bacterial nanocellulose gel were improved, compared to those of the commercially available paper mask. Silk sericin could be control-released from the gel. Furthermore, the silk sericin-releasing bacterial nanocellulose gel was less adhesive and non-cytotoxic, compared to the commercially available paper mask. The *in vivo* safety test confirmed that our silk sericin-releasing bacterial nanocellulose gel was nonirritant. Further investigation into this novel bioactive facial mask has been carried out in clinic. We supposed that the silk sericin-releasing bacterial nanocellulose gel would be an outstanding candidate in the application of facial treatment.

## Methods

### Materials

Coconut water was obtained from coconuts purchased locally. Ammonium sulfate ((NH_4_)_2_PO_4_), glacial acetic acid (CH_3_COOH), and other chemicals used were of analytical grade purchased from Sigma-Aldrich, USA. All chemicals were used without further purification.

### Organism

The *G. xylinus* strain (ATCC 23769) was isolated from *nata de coca* and was supplied by Kasetsart University, Bangkok, Thailand.

### Culture medium

The coconut water sucrose medium as reported in the work of Verschuren *et al.* was used in this study with a slight modification [19]. The initial pH of sucrose medium was adjusted to 3.0, 3.5, 4.0, 4.5, or 5.0 with CH_3_COOH. Then, 10 mL of the *G. xylinus* strain at 10^5^ cfu/mL was added into the pH-adjusted coconut water sucrose medium and poured into tissue culture plates for further fermentation process.

### Fermentation process

For each pH value of the coconut water sucrose medium, the fermentation was incubated at 30°C under static conditions for 7–10 days until the bacterial nanocellulose gels were formed completely. The formed bacterial nanocellulose gels were washed with 2% aqueous NaOH solution at 70°C for 10 min, and then washed repeatedly until a neutral pH was obtained. The bacterial nanocellulose gel was sterilized by gamma radiation before the experiment. Silk sericin solution (0.1 g/mL) was prepared in deionized water. The silk sericin solution (0.2 mL) was absorbed into the bacterial nanocellulose gel (diameter 3.5 cm) and incubated at 25°C for 0.5 h to allow the silk sericin impregnated into the surface of bacterial nanocellulose gel. The silk sericin-releasing bacterial nanocellulose gel was obtained.

### Morphological observation and thickness measurement

The internal structure of samples was observed on a scanning electron microscope (SEM, JSM 5400, JEOL) at an accelerating voltage of 12–15 kV after sputter-coating with gold. The thickness of the bacterial nanocellulose gels prepared from different pH was measured using ImageJ software (the US National Institutes of Health, USA).

### Mechanical test

A tensile test was performed on the wet samples (L = 150 mm, W = 25 mm, H = 3 mm) at room temperature using a universal testing machine (Instron, No. 5567) at a constant rate of 300 mm/min. The force curves as a function of deformation (mm) were automatically recorded by the software. The tensile modulus and elongation at break were calculated according to the ASTM D638-01 method (n = 6).

### Moisture absorption test

The moisture absorption capability of the samples was evaluated by placing the dried sample in the desiccators in which the relative humidity was controlled by salt solution. Potassium chloride was used to obtain a relative humidity of 81.47 ± 1.48% at 25°C [20]. The sample was removed from the desiccators at the predetermined times and carefully weighed. The percentage of weight increase of the sample was calculated relative to their initial weights (n = 6).

### *In vitro* enzymatic biodegradation test

A known weight sample was incubated in 1.6 μg/mL of lysozyme solution (pH 7.4) at 37°C. The enzyme solution was changed every 2 days to ensure continuous enzyme activity. At each time interval, the remaining sample was taken out of the solution, rinsed repeatedly with deionized water, and freeze dried. The dried sample was weighed and the percentage of weight loss was calculated as follows:

1 $$ \mathrm{Percentage}\ \mathrm{of}\ \mathrm{weight}\ \mathrm{loss}\ \left(\%\right) = \left(\frac{W_0-{W}_t}{W_0}\right)\times 100 $$

Where *W*_*0*_ and *W*_*t*_ represent the initial weight of the sample before degradation and the weight of the sample after degradation at different time periods, respectively (n = 3).

### *In vitro* release test of silk sericin

The samples were placed into phosphate-buffered saline solutions (PBS, pH 7.4) at 37°C with a continuous stirring in a closed container. The PBS solutions (1.5 mL) were collected at the predetermined times and the amount of silk sericin released into the solution was measured using a BCA protein assay kit (Pierce, Rockford, IL, USA). The absorbance of the solution was measured at 562 nm and the amount of silk sericin was determined from a standard curve prepared from different concentrations of bovine serum albumin (n = 3).

### Peel test with porcine skin

Porcine skin was used within 2 h after sacrifice. Full-thickness wounds were prepared by cutting the skin to a depth of 1 cm. The samples were attached to the wound. After 4, 24, and 48 h, the sample was removed and the number of cells attached to the sample was analyzed by the fluorometric quantification of cellular DNA according to the method reported by Takahashi *et al.* (n = 3) [21].

### Cytotoxic test with L929 mouse fibroblast cells

A cytotoxic test of the sample was performed using a direct contact assay. High-density polyethylene (HDPE) and natural rubber containing carbon black were used as negative and positive controls, respectively. L929 mouse fibroblast cells (ECACC no. 85011425) were seeded onto the sterilized samples (1 × 1 cm^2^) at a density of 6 × 10^4^ cells/sample and cultured in Dulbecco’s Modified Eagle Medium (DMEM) supplemented with 10% (v/v) Fetal Bovine Serum (FBS) and 100 U/mL of penicillin/streptomycin at 37°C, 5% CO_2_. After 48 h of culture, the cells were stained with 0.01% (w/v) neutral red solution (pH 7.4). The morphology of the cells was observed on a phase contrast microscope.

### Viability test with HaCaT human keratinocyte cells

HaCaT human keratinocyte cells were seeded onto a 96-well plate at a density of 5 × 10^3^ cells/well and cultured in DMEM supplemented with 10% (v/v) FBS and 100 U/mL of penicillin/streptomycin at 37°C, 5% CO_2_. After 24 h of cell attachment, the medium was refreshed and the sterilized sample was placed into the medium. After 3, 6, and 12 h of culture in the presence of the sample, the number of cells was quantified using the conventional 3-(4,5-dimethylthiazol-2-yl)-2,5-diphenyltetra zolium bromide (MTT) assay [22]. The cells cultured on the plate in the presence of a commercially available paper mask in the same conditions were used as a control group (n = 3).

### Cell apoptotic test by Annexin V-FITC/PI double staining assay

HaCaT human keratinocyte cells were cultured in the presence of samples for 3 h, as previously described. Apoptosis-mediated cell death was examined using a double staining method with a fluorescein isothiocyanate (FITC)-labeled Annexin V/propidium iodide (PI) Apoptosis Detection kit (BD Pharmingen™, BD Biosciences, USA) according to the manufacturer’s instructions. Flow cytometric analysis was performed immediately after staining. Cells in the early stages of apoptosis were Annexin-V-FITC-positive, whereas cells that were both Annexin V-FITC and PI-positive were in the late stages of apoptosis.

### *In vivo* safety test (ISO10993-6 Standard)

An *in vivo* test was approved by the Ethics Committee of the Faculty of Medicine, Chulalongkorn University. The animal experiments were performed according to the Chulalongkorn University Animal Care and Use Committee (CU-ACUC) under standard sterile conditions. The implantation of silk sericin-releasing bacterial nanocellulose gel and the commercially available paper mask (2 × 2 cm^2^) into the subcutaneous tissue of female Wistar rats (8 weeks old, 200–300 g) was carried out. Briefly, the rats were anesthetized, and their hair was shaved, and disinfected with 70 vol% ethyl alcohol. A 1 cm skin incision was made to form pockets in the subcutaneous tissue, then the sample was inserted into each pocket. The wound was closed with 6–0 prolene suture and disinfected with Betadine® (povidone-iodine topical antiseptics) solution. After 3, 7, 14, and 28 days of implantation, the rats were sacrificed with an overdose of thiopental sodium. The samples and surrounding tissue were retrieved, fixed with 10 vol% formalin solution, and embedded in paraffin. The paraffin-embedded samples were sectioned and stained with hematoxylin and eosin (H&E). For histological assessment, the H&E slides were semi-quantitatively scored following ISO10993-6. Inflammatory cell types, neovascularization, fibrosis, and fatty infiltrate were scored by one pathologist at two different times. The intensity of inflammatory cells, neovascularization, fibrosis, and fatty infiltrate was recorded using 0–4 scales (0 = not observed, 1 = rare, 2 = minimal, 3 = heavily infiltrated, and 4 = packed infiltrated). The final score was calculated according to Equation 2 and classified as follows: 0.0–2.9 (sample is nonirritant), 3.0–8.9 (sample is slight-irritant), 9.0–15 (sample is moderate-irritant), and >15 (sample is severe-irritant). The level of irritation was compared to that of the commercially available paper mask as a control sample.

2 $$ \mathrm{Final}\ \mathrm{score} = \left[2{I}_t+{N}_t\right]\ \hbox{--}\ \left[2{I}_c+{N}_c\right] $$

where *I*_*i*_ is the total number of polymorphonuclear cells, lymphocytes, plasma cells, macrophages, giant cells, and necrosis of sample *i* (*i* = test sample (*t*) and control (*c*)), *N*_*i*_ is the total number of neovascularization, fibrosis, and fatty infiltrate of sample *i* (*i* = test sample (*t*) and control (*c*)).

### Statistical analysis

All quantitative data represent the mean ± standard deviation. The statistical significance was determined by paired and unpaired Student’s t-tests and ANOVA. A value of *p* < 0.05 was considered significant.
